# The Oncoprotein Mucin 1 in Pancreatic Cancer Onset and Progression: Potential Clinical Implications

**DOI:** 10.3390/biom15020275

**Published:** 2025-02-13

**Authors:** Rosalia Dieli, Rosa Lioy, Fabiana Crispo, Nicoletta Cascelli, Mara Martinelli, Rosa Lerose, Donatella Telesca, Maria Rita Milella, Marco Colella, Simona Loperte, Carmela Mazzoccoli

**Affiliations:** 1 Laboratory of Pre-Clinical and Translational Research, Centro di Riferimento Oncologico della Basilicata (IRCCS-CROB), 85028 Rionero in Vulture, Italy; rosalia.dieli@crob.it (R.D.); rosa.lioy@crob.it (R.L.); nicoletta.cascelli@crob.it (N.C.); mara.martinelli@crob.it (M.M.); carmela.mazzoccoli@crob.it (C.M.); 2 Hospital Pharmacy, Centro di Riferimento Oncologico della Basilicata (IRCCS-CROB), 85028 Rionero in Vulture, Italy; rosa.lerose@crob.it (R.L.); donatella.telesca@crob.it (D.T.); mariarita.milella@crob.it (M.R.M.); 3 Department of Systems Medicine, University of Rome “Tor Vergata”, 00133 Rome, Italy; marco.colella@uniroma2.it; 4 Institute of Methodologies for Environmental Analysis, National Research Council, 85050 Tito Scalo, Italy; simona.loperte@cnr.it

**Keywords:** pancreatic ductal adenocarcinoma, MUC1, tumor microenvironment, EMT, *anoikis*, target therapy

## Abstract

Pancreatic ductal adenocarcinoma (PDAC) is a highly lethal malignancy characterized by poor prognosis, therapeutic resistance, and frequent recurrence. Current therapeutic options for PDAC include surgery, radiotherapy, immunological and targeted approaches. However, all these therapies provide only a slight improvement in patient survival. Consequently, the discovery of novel specific targets is becoming a priority to develop more effective treatments for PDAC. Mucin 1 (MUC1), a transmembrane glycoprotein, is aberrantly glycosylated and frequently overexpressed in pancreatic cancer. Recent studies highlighted the role of this oncoprotein in pancreatic carcinogenesis and its involvement in the acquisition of typical aggressive features of PDAC, like local invasion, metastases, and drug resistance. This review explores the mechanisms by which MUC1 contributes to cancer onset and progression, with a focus on its potential role as a biomarker and novel therapeutic target for pancreatic adenocarcinoma treatment.

## 1. Introduction

Pancreatic cancer is one of the most aggressive and lethal types of neoplastic disorders. Globally it represents the sixth-leading cause of cancer-related deaths [1], with the lowest 5-year relative survival from diagnosis among all solid tumors [2,3,4]. The etiology of pancreatic ductal adenocarcinoma (PDAC) is complex and multifactorial, with cigarette smoking and family history being primary risk factors. Other contributing factors include chronic pancreatitis, age, male gender, type 1 diabetes, obesity, and occupational exposures (e.g., chlorinated hydrocarbon solvents and nickel) [5].

PDAC arises predominantly in the head of the organ, while a lower percentage of neoplastic lesions originate from the body and tail [6]. The disease expands from non-invasive precursor lesions, primarily pancreatic intraepithelial neoplasia (PanIN), which accumulate genetic and epigenetic changes in the course of time. Less common precursor lesions include intraductal papillary mucinous neoplasms (IPMNs) and mucinous cystic neoplasms [7]. Frequently, PDAC is characterized by metastatic spreading, invasiveness, drug resistance, and a high incidence of recurrence [8,9,10].

Histologically, pancreatic cancer displays a heterogeneous tumor microenvironment (TME) and a desmoplastic reaction, both crucial drivers to metastasis formation and chemoresistance [11,12].

Desmoplasia is among the main hallmarks of PDAC. It refers to the accumulation of extracellular matrix components, secreted by both tumor-associated fibroblasts (CAFs) and pancreatic stellate cells (PaSCs) [13]. In particular, fibronectin and collagen (type I and V) are involved in desmoplastic reaction and in processes such as cellular phenotype modification, cell proliferation, intercellular junction development, and invasiveness [14]. Moreover, the dense stromal matrix contributes to generate an hypoxic microenvironment that promotes tumor aggressiveness and drug resistance [15]. A relevant factor, contributing to worse prognoses for PDAC patients, is a late diagnosis due to the lack of specific symptoms in the early phases of the disease and the absence of effective screening tests. Frequently, PDAC patients initiate therapies when the tumor is already at an advanced stage [16,17], with huge limitations in treatment success.

Tumor biology, patient conditions, and disease stage are critical for the treatment choice. Current approaches in PDAC include surgical resection followed by adjuvant chemotherapy and radiotherapy for early stages and palliative chemotherapy. Surgical resection is the only potential curative option, even if just a minority of patients (about 20%) satisfy the eligibility criteria for surgery, such as having a well-localized and resectable tumor and a low preoperative serum level of CA 19.9 [18]. Chemotherapy remains one of the few therapeutic approaches feasible for managing PDAC as well as for treating advanced disease. Chemotherapy regimens include FOLFIRINOX (a combination of 5-fluorouracil, leucovorin, irinotecan, and oxaliplatin) and gemcitabine as a monotherapy or in combination with nab-paclitaxel. Radiotherapy can be used as neoadjuvant or adjuvant treatment in combination with chemotherapy and for symptom control in advanced disease [19]. A late diagnosis and features of aggressiveness and frequent resistance to chemotherapy and radiation highly limit the clinical efficacy of current standard therapies. Instead, the encouraging results, obtained with targeted therapy and immunotherapy for other solid tumors, failed in PDAC because of its peculiar biological characteristics.

Therefore, it is essential to identify new molecular targets, mainly involved in the onset and progression of pancreatic adenocarcinoma, to develop novel therapies for PDAC treatment, both as monotherapy or in combination with standard protocols, in order to improve the life quality and outcome of patients.

Mucin 1 (MUC1), a transmembrane glycoprotein of mucins family, is recognized as an oncoprotein and is usually associated with invasiveness, metastasis, and resistance to therapies in pancreatic ductal adenocarcinoma. During the transition from healthy to neoplastic states, pancreatic cells show alterations in the expression, glycosylation, and localization of the MUC1 protein. In PDAC, MUC1 is overexpressed and hypo-glycosylated and is implicated in activation of several intracellular signaling pathways associated with oncogenesis, proliferation, and tumor dissemination. Indeed, MUC1 regulates the expression of pro-tumoral pathway target genes at transcriptional and post-transcriptional levels.

Exploring the oncogenic multifaced role of MUC1 in pancreatic tumor biology and the impact of its alterations, in terms of expression as well as post-translational modification, in PDAC onset and progression, this review promptly considers MUC1 a potential novel target for innovative drugs in PDAC therapy.

## 2. Structure of MUC1

**Figure 1:**
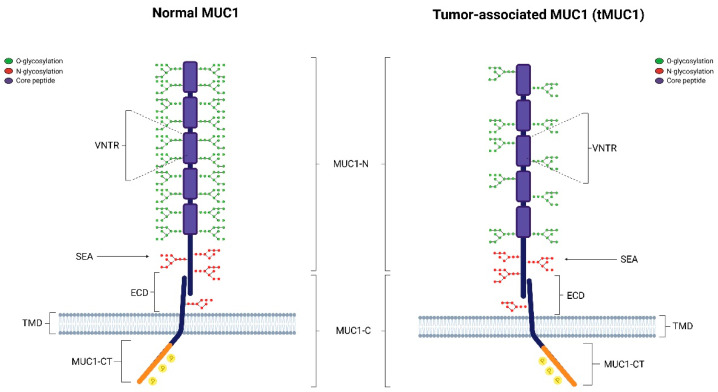
Schematic representation of the MUC1 structure both in physiological conditions (Normal MUC1) and in cancer (tMUC1). MUC1-N = N-terminal domain; MUC1-C = C-terminal domain; VNTR = variable number tandem repeat region; SEA = Sea-urchin sperm protein, enterokinase, and agrin domain; ECD = extracellular domain; TMD = transmembrane domain; MUC1-CT = cytoplasmic tail. In normal cells, O-glycosylation (green) occurs extensively in the VNTR region, while it is reduced in tumor cells. The MUC1-CT contains potential binding motifs for various signaling proteins with phosphorylation sites.

The family of mucins is composed by different types of proteins: trans-membrane (MUC1, MUC4 and MUC16) subtypes, secreted (gel-forming) proteins (MUC7, MUC8, MUC9 and MUC20), and soluble (non-gel forming) mucins (MUC7, MUC8, MUC9 and MUC20). MUC1 is the best characterized mucin member of this family. It is located on the cell apical surface [20] where it becomes involved in several biological processes such as the protection, repair, and survival of the vertebrate epithelia, epithelial self-renewal and differentiation, cell adhesion, and immune response. Structurally, MUC1 is a heterodimer composed of two subunits: the shorter C-terminal domain (MUC1-C) and the longer N-terminal domain (MUC1-N). These two subunits are linked through stable hydrogen bonds [21]. The MUC1 N-terminus is extracellular and consists of a variable number of 20 amino acid tandem repeat (VNRT) whose sequence changes in different cancers. This sequence is abundant in proline, threonine, and serine residues, therefore named the PTS domain. Prolines are responsible for the rigid and inflexible structure of MUC1, while serines and threonines contain O-glycosylation sites. Glycosylated MUC1-N extends above glycocalyx to form a physical barrier with a lubricative function on the mucosal surface, protecting cells from external physiochemical agents. The VNRT region is followed by the sea-urchin sperm protein, enterokinase and agrin (SEA) structural domain. At this level, MUC1 autoproteolysis occurs inducing self-cleavage of the protein. In response to proinflammatory cytokine-mediated signals, such as interferon-γ (INF-γ) and tumor necrosis factor-α (TNF-α), MUC1 is cleaved and the extracellular domain is released from the cell surface into the lumen. The release of MUC1-N activates MUC1-C, which may interact with different target proteins that regulate cellular proliferation, drug resistance, and immune escape. The MUC1 C-terminal domain consists of a 58-amino acid extracellular structural domain (ECD), a 28-amino acid transmembrane structural domain (TMD), and a 72-amino acid cytoplasmic tail (CT) (Figure 1).

Due to its location and numerous interacting proteins, MUC1-C has been widely studied in chronic inflammation and carcinogenesis [22]. The highly conserved cytoplasmic tail of MUC1, composed by several tyrosine, serine, and threonine residues, offers potential docking sites for glycogen synthase kinase-3β (GSK3β), a serine/threonine kinase able to activate a wide range of signal transduction cascades. MUC1-C also regulates Wnt-β catenin, p53, and NF-κB pathways, all linked to cancer progression and drug response.

The glycosylation grade of MUC1 is essential for its functions in normal and cancer cells. In normal cells, MUC1 is heavily glycosylated. Glycosylation stabilizes MUC1 at the cell surface by preventing its clathrin-mediated endocytosis [23]. Indeed, the tumor-associated MUC1 (tMUC1) is characterized by an extensive loss of O-glycosylation. In cancer cells, clathrin-mediated endocytosis increases intracellular intake MUC1 due to its hypoglycosylation. This prevents protein degradation at the intracellular level, triggering MUC1-mediated oncogenic signaling [21].

## 3. MUC1 Role in Cancer Tissues

Precisely for its structure as transmembrane protein across the external and internal cellular environment, MUC1 participates in both outside signaling, sensing the extracellular milieu, and inside transduction pathways, reprogramming the transcriptional profiles of cells in response to the extracellular cues. In this way, it plays an important role in several cellular processes and its dysregulated expression and/or glycosylation may drive carcinogenesis and support the progression of neoplastic cells through the control of immune escape, hypoxic microenvironment establishment, epithelial–mesenchymal transition, and *anoikis*.

### 3.1. MUC1 Promotes Tumor Cells Immune Escape

In PDAC, approximately 90% of the tumor volume is characterized by the presence of a dense and fibrous stroma, capable of contributing to the tumor growth and progression actively [24].

This stroma originates from a desmoplastic reaction in which PSCs and CAFs release a huge amount of fibrotic matrix, inducing the dysregulation of cell-extracellular matrix (ECM) homeostasis and, consequently, promoting the development of cancer and drug resistance [25]. The tumor microenvironment (TME) of PDAC is composed of cellular and acellular components. Immune cells, pancreatic stellate cells, endothelial cells, leukocytes, peripheral nerves, and cancer-associated fibroblasts (CAF) are the cellular fraction of TME and change their function and phenotype in response to the dynamic interaction with the tumor cells. The acellular fraction includes ECM proteins, chemokines, cytokines [26], and a wide range of growth factors, such as fibroblasts growth factors (FGFs), epidermal growth factors (EGFs), nerve growth factors (NGFs), transforming growth factors β (TGFs-β) isoforms, and connective tissue growth factors (CTGF). Frequently, the TME of pancreatic cancer also encloses an elevated quantity of myeloid-derived suppressor cells (MDSCs) and T regulatory cells (Tregs), which repress the tumor-specific immune response, and minor quantities of tumor infiltrating lymphocytes (TILs), whose function is to identify and attack cancer cells [27]. The immunosuppressive and desmoplastic characteristics of the microenvironment give pancreatic tumor cells the ability to escape the mechanisms of immunosurveillance, enjoying the so-called immunological privilege [28].

On this basis, the distinctive feature of PDAC is a heterogeneous organization, which leads to a dynamic composition characterized by a cross-talk between the tumoral cells and stromal components that affects the efficacy of a chemotherapeutic protocol and the overall survival [29].

In pancreatic cancer, MUC1 may interact and regulate different components of the TME, supplying various processes such as neoangiogenesis, metastasis, immune evasion, and oncogenic signaling. Due to its large size, in physiological conditions, MUC1 acts like a protective barrier against pathogens, providing a protective shield on the cell surface and inhibiting the possible cell–cell or cell–ECM interactions [30]. On the other hand, several studies demonstrated the immunosuppressive effect of MUC1. In pathological conditions, MUC1 covers the surface of tumor cells and protects them from the cytotoxic components of cell-mediated immunity, masking tumor-associated antigens TAAs [31].

In this regard, the high affinity interaction between Galectin-3 (Gal-3), a member of β-galactoside binding protein family, and T antigen, located on the MUC1 [32], is fundamental for TME regulation and cancer [33]. Gal-3 is present in blood as well as both inside and outside of cells. While extracellular Gal-3 acts as an adhesion molecule in cell–cell interactions and facilitates the growth and spread of cancer cells, intracellular Gal-3 inhibits apoptosis and promotes mRNA splicing [34]. Serum levels of free circulating Gal-3 are significantly higher in patients with several solid tumors, including PDAC [35]. Furthermore, Gal-3 serum levels are higher in patients with metastatic disease compared to localized tumors.

Structurally, Gal-3 presents an extended N-terminal domain and a highly conserved β-galactoside-binding domain which binds the T antigen, a type 1 O-glycan linked with the Ser/Thr residues of MUC1, with immunotherapeutic properties. A significant consequence of the interaction between the Gal-3 and the T antigen on the MUC1 extracellular domain is the mucin clustering and polarization on the cell surface, which reveals and makes available that smaller cell surface adhesion molecules, such as E-cadherin (E-Cad), previously masked by glycosylated MUC1. This may facilitate the binding of tumor cells to endothelial cells, supporting metastatic process [33]. In addition, modifications of MUC1 cell surface localization, in response to Gal-3 binding, cause homotypic aggregation of cancer cells and the development of circulating tumor emboli, thereby avoiding *anoikis* and extending the duration of neoplastic cell survival [35].

Due to their implications in the processes of progression and metastasis, MUC1/T antigen-Gal-3 binding represents a fascinating target for new therapeutic strategies based on the inhibition of this interaction [36].

### 3.2. MUC1 Influences Hypoxic Tumor Microenvironment

Hypoxia is a common condition of tumors in which oxygen’s request for tissue is unsatisfied. It results from aberrant tumor vascularization, commonly linked to the massive growth of solid tumors. In order to maintain cell growth and survival, rapidly growing tumors adapt to hypoxic environment through either angiogenesis, which ensures an adequate supply of oxygen and nutrients through neo-vascularization, or migration and metastasis, which allow to shift towards a more comfortable microenvironment [37].

The frequent presence of a hypoxic microenvironment in malignancies, including pancreatic ductal adenocarcinoma, is tightly linked to the overexpression of hypoxia-inducible factor-1 alpha (HIF-1α), a key transcriptional factor in cancer cells able to regulate the expression of many genes in the organism when stabilized by hypoxia [38].

Further studies have demonstrated that HIF-1α is associated with a poor prognosis due to its multiple contribution in interfering with the response to targeted therapies, chemotherapy, and radiotherapy, but also because of the involvement of its target genes in numerous pro-tumoral pathways, such as angiogenesis, immune escape, invasiveness, cell proliferation, survival, and glucose metabolism [39,40].

**Figure 2:**
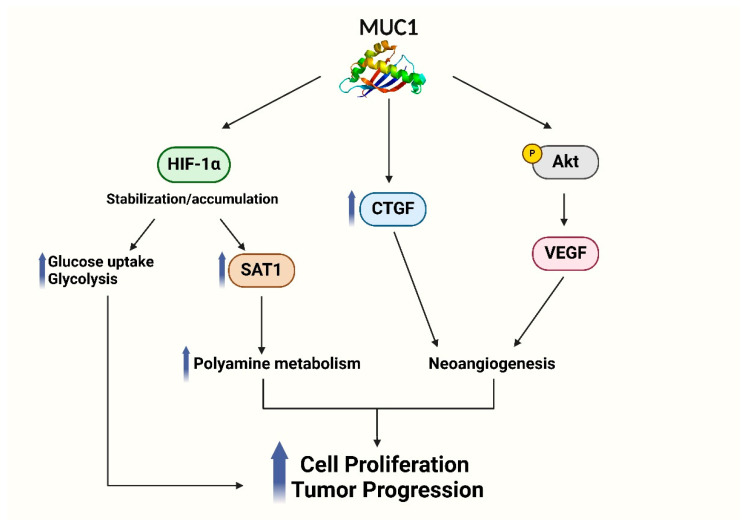
Schematic representation of MUC1 role in hypoxic signaling. MUC1 induces HIF-1α stabilization and accumulation, with a consequent upregulation of glycolysis and polyamine metabolism through SAT1 overexpression. MUC1 regulates the transcription of hypoxia-related genes *CTGF* and *VEGF*, which contribute to neoangiogenesis in pancreatic cancer cells. Hypoxic signaling promotes a more aggressive phenotype, supporting proliferation and tumor growth under low oxygen conditions.

The expression of MUC1 in cancerous tissues exhibits a substantial correlation with markers associated with hypoxia, including HIF-1α, vascular endothelial growth factor (VEGF), and Ki-67 labeling index [41] (Figure 2).

In pancreatic cancer cells, MUC1 expression increases in a time-dependent manner under hypoxic conditions. Moreover, the response is higher in metastatic cell lines, such as AsPC1, compared to primary cancer lines, such as BxPC3. In particular, the nuclear translocation of HIF-1α is favored in order to regulate the hypoxia-related genes like *CTGF* or *VEGFA* and contribute to neoangiogenesis [42].

Bhernes and colleagues demonstrated that MUC1 induces expression of *CTGF* gene by phosphorylation events on the MUC1-CT. CTGF, a direct transcriptional factor of HIF-1α, is a strong angiogenetic factor induced by hypoxia in several cancers. Specifically, CTGF acts like a growth factor in inflammation, cell adhesion, tumor growth, and fibrosis processes [43].

It has also been demonstrated that under hypoxic conditions, MUC1 may influence VEGFA downstream effects. Specifically, MUC1 is able to promote the synthesis and secretion of VEGF, a potent angiogenic factor, by activating the AKT signaling pathway [44].

MUC1 may also regulate metabolism in pancreatic cancer cells interacting with HIF-1α. In fact, its expression activates the transcription of glycolytic genes, increasing the glucose uptake and consumption in PDAC cells. This effect is observed mainly under hypoxic conditions, underlining the important correlation between the presence of HIF-1α and the regulation of cell metabolism in promoting metabolic alterations which help tumor cells to survive and proliferate under unfavorable environments [45].

The MUC1-HIF-1α signaling pathway is also able to regulate polyamine metabolism, a key survival pathway in cancer cells. Polyamines play a pivotal role in numerous crucial processes of cell proliferation, such as protein and nucleic acid synthesis, chromatin structure stability, cell differentiation, induction of apoptosis, protection against oxidative damage, and control of various ion channels essential for intercellular communication. Moreover, polyamines are essential for controlling immunological response, since they are able to modulate the activity of immune cells such as T cells, B cells, macrophages, and natural killer cells [46,47].

Murthy et al. demonstrated a positive correlation between MUC1 and spermidine/spermine N1-acetyltransferase (SAT1) expression, a crucial enzyme implicated in polyamine metabolism. Specifically, through the stabilization of HIF-1α, MUC1 increases SAT1 expression, which leads PDAC cells to change their metabolic state to oxidative phosphorylation. Furthermore, HIF-1α inhibition decreases SAT1 expression and limits cell proliferation in hypoxic conditions, demonstrating that HIF-1α and SAT1 are cross-regulated to maintain polyamine homeostasis in PDAC cells [48].

### 3.3. MUC1 Promotes Epithelial to Mesenchymal Transition

The epithelial-to-mesenchymal transition (EMT) is a paradigm of cellular plasticity implicated in embryonic development, tissue regeneration, and tumorigenesis [49]. EMT involves a cellular phenotypic switch regulated by a network of specific transcription factors (EMT-TFs), including Snail Family Transcriptional Repressors 1 (Snail1) and Snail2, Twist-related protein 1 (Twist1), and Zinc Finger E-box-binding Homeobox 1 (ZEB1) and ZEB2. These EMT-TFs are responsible for the down-regulation of epithelial markers and the up-regulation of mesenchymal ones, such as vimentin and N-cadherin (N-Cad) [50].

In the tumor context, EMT significantly contributes to increasing migration and invasion of cancer cells into surrounding tissues, primarily due to the transcriptional inhibition and functional loss of E-cadherin (E-Cad), a key protein involved in epithelial differentiation and formation, stabilization, and proper functions of adherens junctions [51]. Thus, EMT is part of the metastatic cascade that facilitates cancer spread.

**Figure 3:**
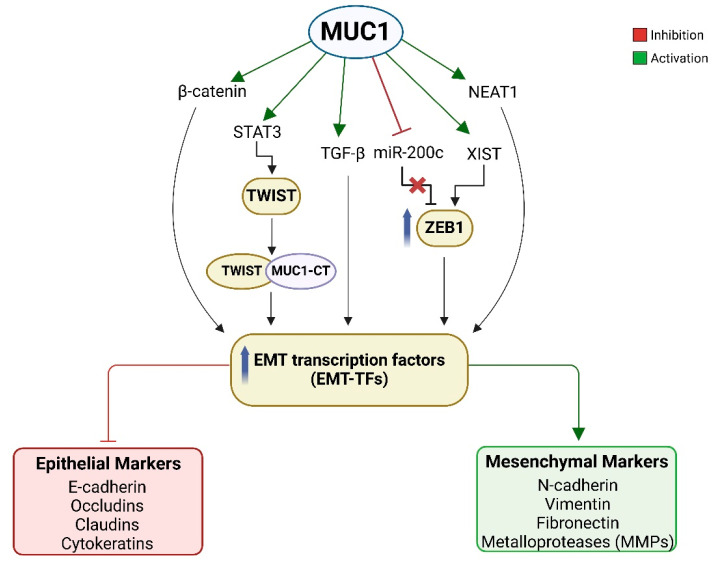
Schematic representation of MUC1 role in epithelial to mesenchymal transition (EMT). MUC1 activates EMT through interactions with β-catenin, STAT3, TGF-β, and lncRNAs XIST and NEAT1, leading to the upregulation of EMT transcriptional factors. Consequently, the induction of mesenchymal markers occurs, while epithelial markers are downregulated. MUC1 also mediates miR-200c transcriptional repression, which results in ZEB1 overexpression and consequent EMT promotion.

In PDAC, EMT strongly correlates with cancer progression, metastasis, a high incidence of recurrence, and chemotherapy resistance, and this linking partially explains the poor prognosis of pancreatic tumors [52,53]. In vitro experiments on pancreatic cancer cells showed that the reversion of EMT process enhances the sensitivity of cancer cells to gemcitabine [54]. MUC1 is capable of activating EMT by direct interaction with principal actors of phenotype transition (Figure 3).

In vivo experiments demonstrated a lower invasiveness and ability to give metastasis in a PDAC mouse model of MUC1 knockout (PDAC.MUC1KO). MUC1 expression in PDAC.MUC1 mice correlates with a significant increase in Snail, Vimentin, and Slug expression, suggesting the ability of MUC1 to trigger EMT process [55]. Further studies established that the cytoplasmic tail of MUC1 is crucial to initiate the process of EMT in several carcinomas, including PDAC. In human pancreatic cell lines BxPC3 and Su86.86, transfected with mutated MUC1-CT for all the seven tyrosine residues, EMT is significantly reduced when compared to control cells with non-mutated MUC1-CT. Indeed, tyrosine residues phosphorylation enables MUC1-CT to associate with β-catenin and translocate to the nucleus, leading to the upregulation of EMT-TFs and consequent repression of E-Cad at both transcriptional and protein levels. This demonstrates the MUC1 role in controlling the Wingless-related integration site (Wnt) signaling cascade. Moreover, the promotion of nuclear localization of the MUC1-CT/β-catenin transcriptional complex is facilitated by platelet-derived growth factor β (PDGF-β) stimulation and overall raises the potentiality of PDAC cell invasion [56,57].

Several studies showed that *MUC1* gene promoter presents a responsive region for signal transducer and activator of transcription 3 (STAT3), a transcriptional factor with a crucial role in regulating the expression of genes associated with cell differentiation, proliferation, inhibition of apoptosis, and angiogenesis [58,59]. STAT3 is constitutively activated in PDAC cell lines, PDAC xenografts, and primary human PDAC, playing a role in tumor development and EMT activation [60,61,62,63]. MUC1 and STAT3 interaction results in an auto-inductive loop in which MUC1-CT directly binds to both cytokine receptor-associated Janus kinase 1 (JAK1) and STAT3, facilitating STAT3 activation via JAK-1-mediated Tyr705 phosphorylation. The final effect is the transcription of *MUC1* gene by active STAT3. Therefore, targeting STAT3 and MUC1 interplay may be a strategy for enhanced anti-tumor efficacy. As a matter of fact, in a recent study Bose et al. demonstrated that both human and murine PDAC cell lines, expressing high MUC1 levels, are more sensitive to the STAT3 inhibitor Napabucasin compared to cancer cells with low MUC1 expression, confirming the synergistic interaction between MUC1-CT and STAT3 in vitro [64]. Furthermore, STAT3 activation leads to the transcriptional induction of TWIST1, which in turn binds directly to MUC1-C. Eventually, the MUC1-CT/TWIST1 complexes activate MUC1-CT expression in an auto-inducible cycle. This circuit is sufficient for driving the expression of multiple EMT-program related genes, such as ZEB1 and SNAIL, with the consequential loss of epithelial cell–cell adhesion and the acquisition of an invasive phenotype of cancer cells [65].

The tumor growth factor β (TGF-β) is a member of a superfamily that includes bone morphogenetic proteins (BMPs), known to act as tumor-suppressor at early stages and oncogenic protein in the later stages of cancer development. Among all isoforms, TGF-β1 is probably the main EMT promoter in pancreatic cancer, as supported by several in vitro observations in human pancreatic cancer cell lines [66,67,68]. Clinical-pathological investigations in patients with PDAC demonstrated the correlation between the plasma levels of TGF-β1 and advanced tumor stage, metastasis, and dismal survival [69,70,71]. The interaction between MUC1 and TGF-β1 has been investigated, revealing a synergistic association in promoting EMT and proliferation. Specifically, MUC1 regulates the TGF-β1 function switching from a tumor-suppressor to a tumor-promoter through phosphorylation of its C-terminal tyrosines independently by SMAD4 signaling. This process is fundamental for downstream signal transduction like TGF-β-associated apoptosis and invasion [72]. MUC1- TGF-β axis has interesting clinical implications since PDAC patients with MUC1 overexpression may be candidates for anti-TGF-β target therapies. In this regard, treatment with anti-TGF-β neutralizing antibody leads to a significant reduction in the tumor growth of xenograft mice models characterized by high-MUC1 neoplasia [73].

It is well known that non-coding RNAs (ncRNAs), such as microRNAs (miRNAs) and long-non-coding RNAs (lncRNAs), are also involved in the regulation of EMT [74,75,76]. MiRNAs are single-stranded RNA molecules with a length of about 22 nucleotides that exert post-transcriptional gene silencing. LncRNAs are around 200 nucleotides strands which provide a variety of purposes for other molecules, including acting as tethers, scaffolds, decoys, and guides [77]. The altered expression of ncRNAs plays a determinant role in the shaping of tumor nature [78].

The miR-200 family is widely studied in cancer because the overexpression of its individual miRNA members or their clusters is implicated in EMT repression through targeting the transcripts of numerous genes involved in this process [79,80]. miR-200c is one of the most studied members of the miR-200 family. Its loss correlates with the maintenance of stemness and the lack of E-Cad expression in invasive pancreatic cancer cells and in pancreatic cancer specimens, supporting the repressive role of miR-200c on EMT process [81,82]. Indeed, ZEB1 and miR-200c interact and regulate the EMT process in opposite manners, forming a negative feedback loop [83,84,85]. Considering that both MUC1 and miR-200c are associated with cancer progression and EMT, some authors investigated a possible correlation between them. A study conducted on S2.013.MUC1 and Panc1.MUC1 pancreatic cells demonstrated that miR-200c expression negatively correlates with MUC1. In particular, MUC1-CT directly complexes with ZEB1 and binds the promoter of the miR-200c/141 cluster, resulting in miR-200c transcriptional repression. MiR-200c loss carries ZEB1 upregulation along with consequent EMT promotion due to ZEB1-mediated E-Cad down-regulation [86].

MUC1-CT is also capable of influencing the activity of some lncRNAs, such as X inactive-specific transcript (XIST) and Nuclear Enriched Abundant Transcript 1 (NEAT1). The first is a major factor in female X chromosome inactivation and is encoded by the *XIST* gene [87]. XIST is dysregulated in PDAC and is deeply involved in the metastatic cascade. In fact, XIST-knockdown pancreatic adenocarcinoma cells show a decrease in migration, invasion, and EMT capacities [88,89]. Further, mechanistic exploration demonstrates the existence of an oncogenic XIST/miR-429/ZEB1 axis that drives pancreatic cancer progression [90].

MUC1 is able to control XIST lncRNA at different levels. A recent study demonstrated that the MUC1-CT reduces the m6A methylation complex components that bind XIST, specifically RBM15/B, WTAP, and METTL3/14, avoiding the control of a stable silencing of X-linked genes [91]. Additionally, MUC1-CT inhibits the YTHDF2-CNOT1 de-adenylase complex, which is capable of identifying m6A sites fundamental for addressing XIST degradation [91]. The result is an increase in XIST stability and, consequently, XIST lncRNA expression level. On the other hand, it was demonstrated that XIST may regulate MUC1-CT expression by promoting NF-κB-mediated activation of the MUC1 gene.

Previous studies have observed a positive correlation between expression levels of NEAT1 and the progression of PDAC, along with worst overall survival [92]. NEAT1 serves as a scaffold for nuclear RNA-binding proteins to form the *paraspeckles*, ribonucleoprotein bodies, found in the interchromatin space of mammalian cell nuclei. *Paraspeckle* forms by the aggregation of NEAT1 lnc-RNA with multiple RNA-binding proteins, secondary to particular environmental stimuli, and influences various cellular processes, including transcription, RNA splicing, and cellular stress response [93]. It is known that the downregulation of NEAT1 enhances the sensitivity of pancreatic adenocarcinoma cells to gemcitabine by reversing the EMT process [94]. A group of researchers examined the role of the MUC1-CT protein as a positive regulator of NEAT1 lncRNA expression. Consequently, *paraspeckle* formation in neoplastic cells may depend by MUC1-CT expression. The development of ribonucleoprotein bodies is promoted in MUC1 high-level cellular background, while it is reduced in knockdown models. Furthermore, NEAT1 is essential for the stability of the MUC1-CT protein, suggesting that NEAT1 probably helps to maintain the oncogenic activity of MUC1-CT in cancer cells. The study also highlighted that this co-regulation promotes gene signatures linked to inflammation and EMT, such as interleuchin-6 (IL-6) and dual phosphatase (DUSP2), facilitating cancer cell dissemination and metastasis formation [95]. Therefore, the regulation of NEAT1 and *paraspeckles* formation indicates a novel mechanism by which MUC1-CT may influence cancer biology, suggesting the potentiality of targeting these signaling pathway for future treatments in PDAC.

### 3.4. MUC1 Promotes Anoikis

*Anoikis* represents a type of programmed cell death caused by inadequate contacts between the cells and ECM and loss of cell–cell adhesion, hampering re-adhesion in inappropriate sites of detached epithelial cells. Within a normal microenvironment, specific integrins can recruit focal adhesion kinase (FAK), integrin-linked kinase (ILK), and phosphatidylinositol 3 phosphate kinase (PI3K) to activate the EGFR/PI3K signaling pathway, which regulates cell proliferation as well as inhibits *anoikis*. In contrast, when cell adhesion is lost and integrin disengagement occurs, the *anoikis* process starts as a result of the ERK signaling suppression with consequential activation of pro-apoptotic proteins and a decrease in anti-apoptotic protein levels [96].

**Figure 4:**
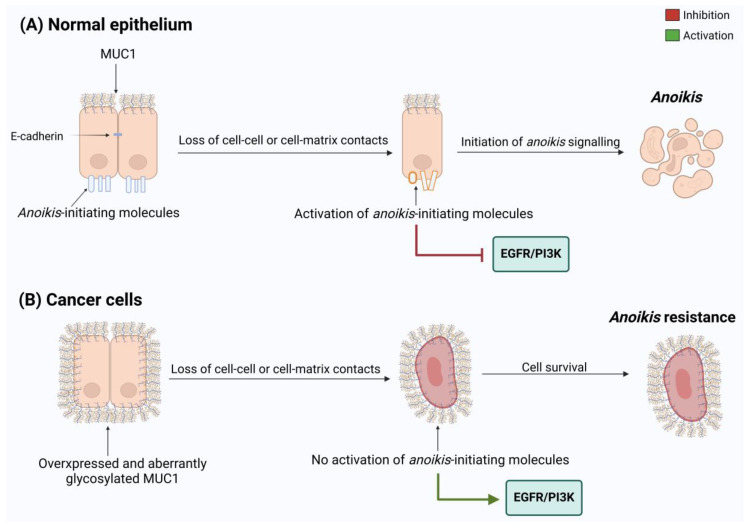
Influence of MUC1 expression levels on *anoikis*. In normal epithelial cells, the loss of the cell–cell- and/or cell-ECM contact activates *anoikis* by inhibition of EGFR/PI3K pathway (red line). In cancer, MUC1 is overexpressed and covers the entire cell surface, interacting with *anoikis*-initiating molecules, preventing their activation. So, the EGFR/PI3K pathway results are hyper-activated (green line) and sustain *anoikis*-resistance.

*Anoikis* may take place through either the intrinsic or extrinsic apoptotic pathway: the first occurs by the release of cytochrome *c* from mitochondria, while the second is mediated by death receptors signaling. Both ways culminate with caspase activation, DNA breakage, and apoptotic body formation. Moreover, *anoikis* can be induced apart from caspase activity by the release of the mitochondrial mediator Bit-1 into the cytoplasm after the loss of integrin attachment [97]. *Anoikis* resistance is emerging as a new hallmark of cancer and may have a prognostic value. Resistant cells gain the ability to survive and grow in the absence of an attachment to the ECM, allowing them to migrate in the blood circulation and colonize distant organs [98]. The ability to overcome *anoikis* may result from the acquisition of the mesenchymal phenotype, since most EMT-TFs are able to modulate pro- and anti-apoptotic genes [99,100]. In PDAC, one of the fundamental mechanisms involved in the acquisition of *anoikis* resistance is the activation and overexpression of STAT3. Specifically, AsPc-1, Panc-1, HPAC, L3.6PL, and COLO-357 metastatic cell lines show a reduction in *anoikis* resistance and fail to form tumors and metastasize in vivo after treatment with the STAT3 inhibitor AG 490 [101]. Other important mechanisms include the hyper-activation of the EGFR/PI3K pathway, cell aggregate formation, and metabolic rewiring [102]. The expression of several *anoikis*-related genes has been found to correlate with the prognosis of pancreatic ductal adenocarcinoma (PDAC). A recent study identified four *anoikis*-associated genes—ITGA3, CDK11A, RHOG, and TNFSF10—and used them to develop a prognostic model. Indeed, high levels of ITGA3 and TNFSF10 are associated with a poorer prognosis, whereas CDK11A and RHOG act as protective factors [103]. Considering that MUC1 exerts a prominent role in PDAC progression, and its overexpression correlates with metastatic cancer and poor prognosis, several studies were conducted to elucidate the MUC1 involvement in regulation of *anoikis*. According to Piyush et al., MUC1 expression and O-glycosylation correlate with *anoikis* resistance. Indeed, the inhibition of enzymes involved in the production of O-linked mucin-type glycans, such as Core 1 Gal-transferase (C1GalT), leads to *anoikis* and interrupts the start of metastatic processes [104,105] (Figure 4).

MUC1 large O-glycosylation significantly inhibits the accessibility of ligands of *anoikis*-initiating molecules such E-Cad, Fas, and integrin β to cell surfaces, hence causing resistance to *anoikis*, and, consequently, the loss of cell adhesion [105]. Related studies demonstrated that adhesion molecules are exposed subsequently because of MUC1 polarization on the cell surface, which is mediated by Gal-3 interaction. This event implies increased homotypic tumor cell adhesion and causes anchorage-independent cells to avoid *anoikis* [105,106]. A recent study demonstrated the efficacy of the TAB004 monoclonal antibody, specifically for the aberrantly glycosylated form of MUC1 (tMUC1), in reducing significantly EGFR phosphorylation and inducing MUC1 degradation in PDAC, both in vitro and in vivo. The authors hypothesized that TAB004 binding with extracellular MUC1-N prevents the interaction of EGF with EGFR due to a steric hindrance of antibody-target complex on cell surface. This interference results in the inhibition of the EGFR-PI3K pathway, thereby suppressing *anoikis* resistance both in vitro and in vivo. Additionally, a notable decrease in the binding of MUC1 to desmosomal proteins is observed following TAB004 treatment. The impairment of MUC1-desmosomal interaction reverses *anoikis* resistance, also impairing cancer cell ability to form colonies and adhere to the ECM and/or neighboring cells [107].

## 4. MUC1 as Prognostic Marker for Target Therapies

Pancreatic cancer is one of the most challenging cancers to treat due to its multifactorial nature and the entangled, highly vascular and fibrous surrounding environment, which make surgical removal difficult as well as chemotherapy and immunotherapy treatments ineffective. Moreover, the diagnosis arrives too late because of symptoms overlap with other common ailments, and image diagnostics hardly for detecting small lesions [108].

In recent years, the number of deaths, incidences, and disability-adjusted life-years (DALYs) caused by pancreatic cancer has doubled. Thus, the discovery of new diagnostic and treatment strategies is becoming a healthy emergency for society [109].

The early diagnosis of cancer is important for improving treatment success and survival rates. In cancers with poor prognosis, like pancreatic cancer, there are no diagnostic biomarkers or screening programs to recognize the disease at earlier stages or to identify candidates for specific treatments [110]. Due to MUC1’s aberrant expression in cancer epithelial cells, MUC1 recently raised scientific interest as a potential prognostic and/or therapeutic biomarker [111].

PDAC is one of the best examples of cancer for which the amount of mucin, as well as the polarization and pattern of mucin glycosylation, may allow a better pathological classification, diagnosis, and prognostic evaluation of this neoplasia. In fact, MUC1 expression in normal pancreas cells begins to change concurrently with early genetic and molecular events, which lead to precursor lesions and tumor progression afterwards [112]. Several diagnostic methods were proposed to detect MUC1 concentration with high sensitivity and specificity. The most sensitive approaches used for early detection of mucins include surface-enhanced Raman spectroscopy (SERS), gold magnetic nanorod immunoassays, electrochemiluminescence, and PET scans [113]. Among them, the colorimetric immunoassay using magnetic gold nanobonets demonstrated the best performance in terms of ease of use, sensitivity, and specificity for MUC1 detection in a small volume of serum sample [114].

**Figure 5:**
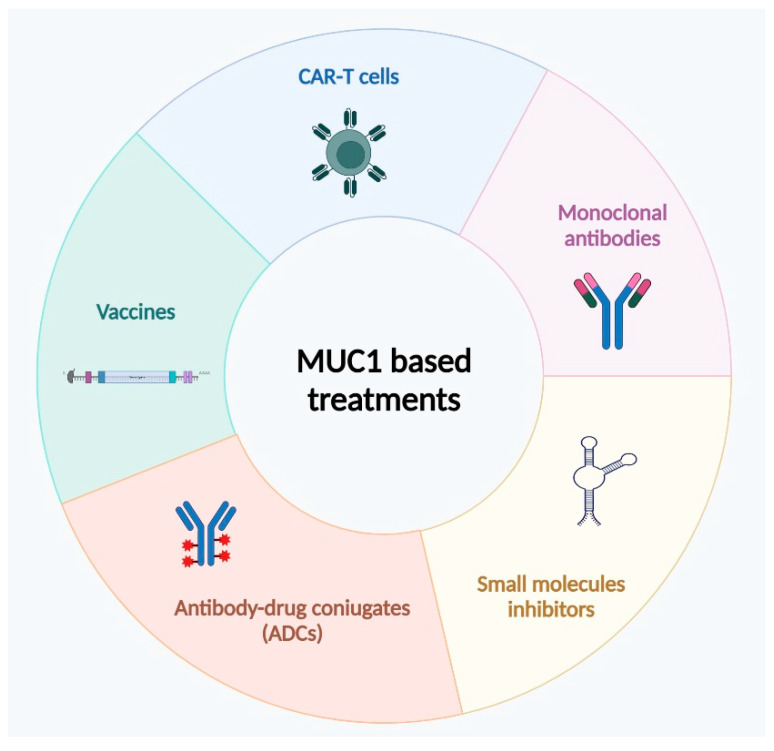
Therapeutic applications of MUC1 in PDAC. Small molecule inhibitors usually inhibit MUC1-CT. Monoclonal antibodies target both MUC1-N and MUC1-C, reducing tumor growth and metastasis, while antibody-drug conjugates (ADCs) deliver selectively cytotoxic agents to cancer cells. MUC1-based vaccines and CAR-T cell therapy aim to induce an immune response against MUC1-expressing tumor cells.

The progression in the development of analytical methods for the evaluation of mucin levels in the serum of oncological patients should help the use of MUC1 pattern expression/polarization as a therapeutic marker for clinical applications of MUC1-targeted innovative therapies (Figure 5). Indeed, PDAC patients have very limited treatment options, often palliative, and only one-fifth of them receive a tempestive diagnosis to try surgery followed by adjuvant chemotherapy, a unique available approach with curative intent. So, the research of innovative and personalized therapies represents a a huge gap for oncologists.

Chemotherapy regimens usually involve the administration of single or multidrug combination of gemcitabine and fluorouracil. Unfortunately, patients begin to show resistance to therapy a few weeks after treatment starts. Due to the strong evidence regarding the correlation between MUC1 and drug resistance in malignant tumors, MUC1 became the promising target of new therapeutic strategies based on the inhibition of its expression or functions, which could improve PDAC patient responses to conventional therapies. Treatments targeting MUC1 can be classified into three groups: vaccine inducing an immune response to MUC1, antibodies targeting MUC1-N or -C terminal domain, and MUC1 inhibitors.

### 4.1. Immunotherapeutic Approaches

Due to diagnoses in advanced stages of PDAC, immunotherapy has not been particularly studied as an alternative treatment for this tumor. MUC1, however, was found to be a potential anti-neoplastic antigen because of its change in the expression profile of normal and cancer cells, and it has been identified by the American Cancer Institute Working as one of the most promising cancer vaccine-targeted antigens in clinical trials [21]. For targeting MUC1, several vaccines based on different technologies (DNA, viral vectors, subunits, dendritic cell, and glycopeptides) were developed and are already in clinical trials [115].

The B subunit of the Vibrio cholerae toxin (CTB) has proven to be an excellent vector for subunit vaccines. Pinkhasov et al. evaluated the effects of administering the CTB-MUC1 vaccine on mice with pancreas adenocarcinoma and observed an effective inhibition of the tumor growth [116]. Other studies have shown that the administration of different vaccine types enhances dendritic cell (DC) antigen presentation and activates cytotoxic T cells [117,118]. Some experiments on mouse model and a few clinical trials on pancreatic cancer and other solid tumors demonstrated that DCs vaccines loaded with MUC1 show an effective durable response with good safe and tolerability [115]. Moreover, antitumor vaccinations, based on MUC1 glycosylated tricomponents, clearly reduced the tumor burden by stimulating humoral and cellular immune responses [119].

In addition to the use of vaccines, another immunotherapy approach for PDAC treatment is based on chimeric antigen receptor T cells (CAR-T). CAR-T cells are recombinant antigen receptors that modify the function and specificity of T lymphocytes and other immune cells. Their application in cancer immunotherapy is based on the fundamental concept that they can quickly produce tumor-targeted T cells, avoiding the obstacles and gradual kinetics of active immunization. CAR-T cells have shown a huge potential for curing hematologic cancers, while the clinical application in solid tumors is limited since the tumor microenvironment inhibits their efficacy [120]. Nevertheless, some researchers recently engineered CAR-T cells using the variable fragments of a novel monoclonal antibody, TAB004, which specifically binds the tumor-associated-MUC1 (tMUC1) to PDA cells, showing promising cytotoxic activity [121].

Because of the high concentration of tumor-associated macrophages (TAMs) in solid tumor microenvironments, CAR-M cells have been developed as an alternative therapy to CAR-Ts. The first-generation CAR-Ms recognize tumor cells and enhance their phagocytosis by targeting particular antigens. Second-generation CAR-Ms improve also T-cell activation and tumor antigen presentation [122]. Based on the results of these studies, the combined administration of CAR-T/CAR-M as an adjuvant treatment could represent a promising therapeutic strategy in the treatment of PDAC.

### 4.2. Antibodies

Antibody-based therapies are widely used for the treatment of solid tumors due to their specificity and binding affinity. Several therapies, based on neutralizing anti-bodies, chimeric antigen receptors, bispecific T-cell binding, and antibody-drug conjugates, were developed over the years to target the extracellular domain of MUC1.

Monoclonal antibodies (mAb), nanoparticle antibodies, and bispecific antibodies (bsAb) are employed as new therapeutic strategies. PAM4 is a murine mAb obtained by immunizing mice with human pancreatic cell-derived mucin. PAM4 is characterized by high specificity for pancreatic ductal adenocarcinoma and for its precursor lesions, and it is able to discriminate between PDAC and non-neoplastic tissues of the pancreas. For its capacity to detect early-stage and advanced stages of disease, PAM4-based immunoassay is employed for evaluation of tissues specimens and for detection of PDAC [123].

TAB004, a humanized IgG1 mAb, identifies PC cells in patients with stage II-IV of PDAC, but it is unable to bind healthy cells. TAB004 reverses apoptosis resistance MUC1-mediated, by means of mucin degradation. In a PDA xenograft, the treatment with this mAb reduces tumor growth and increases survival in mice treated with 5-FU combination compared to IgG controls [124]. KL-6, a MUC1-derived glycoprotein antigen, is a mouse IgG1 mAb that recognizes KL-6 sialic acid. The 99mTc-labeled KL-6 is a tumor-specific radioactive tracker for PC used in vivo for pancreatic cancer detection specifically for this characteristics [124].

### 4.3. MUC1 Inhibitors

GO-201 is a peptide targeting MUC1-CT CQC motif acting as inhibitor of cytoplasmic tail oligomerization. In in vitro prostate cancer models, GO-201promoted cell death, while, in prostate tumor xenograft models, its use supported complete regression. Additionally, the inhibitor GO-201 showed marked reduction in viability and proliferation of pancreatic cancer cells, as well as significant decrease in tumor burden in mouse model of PDAC [125]. Considering this evidence, MUC1-CT cytoplasmic domain configured as a good target for development of new drug inhibitors.

## 5. Conclusions

Pancreatic adenocarcinoma is an aggressive epithelial malignancy characterized by high rates of incidence and mortality. An important correlation exists between pancreatic adenocarcinoma and MUC1, a glycoprotein of the mucin family. In patients with locally advanced or metastatic disease, overexpression and altered glycosylation of MUC1 are frequently observed. In healthy epithelial cells, MUC1 is normally glycosylated and forms a protective barrier from stress-induced damage with N-terminal domain. Instead, in tumor tissues, MUC1 is aberrantly expressed and glycosylated and its localization is altered. These aspects confer MUC1 the features of an oncogene. In PDAC, its C-terminal subunits are involved in the activation of signaling pathways correlated with progression, metastasis, and invasion. Moreover, interacting with HIF-1, MUC1 induces a metabolic reprogramming responsible for gemcitabine chemoresistance and hypoxic tumor microenvironment.

In the serum of pancreatic cancer patients, MUC1 levels increase in a stage-dependent manner and so MUC1 expression may be potentially used as biomarker for the diagnosis and staging of PDAC patients. Furthermore, since cancer cells release MUC1, its measurement is very important as it allows us to monitor the clinical response of patients to therapy. In addition to playing an important role in the field of cancer biomarkers, MUC1, promoting PDAC carcinogenesis, represents a good therapeutic target for new opportunities for cancer treatment.

Therapeutic treatments targeting MUC1, such as vaccines, antibodies, and CAR-T focus on breakthroughs in immune response and chemoresistance and represent an important research trajectory in order to improve individual response to treatments. The numerous clinical trials activated in the last ten years demonstrate how the preparation of anti-tumor drugs against MUC1 is of great importance for the comprehensive treatment of pancreatic cancer. Despite MUC1 involvement in many biological processes of PDAC carcinogenesis, it is improbable that inhibition of MUC1 alone may arrest the tumor progression. Probably the optimal therapy for PDAC patient management could consist of combined standard-of-care approaches with novel agents that target MUC1 for more effective personalized anticancer treatments.

## Data Availability

All data generated or analyzed during this study are included in this published article.
